# In vitro evaluation of enterococcus faecalis growth in different conditions on dentinal substrate

**DOI:** 10.1080/26415275.2023.2287668

**Published:** 2023-12-20

**Authors:** Wajih Hage, Dolla Karam Sarkis, Mireille Kallassy, May Mallah, Carla Zogheib

**Affiliations:** a Departement of Endodontics, Saint Joseph University, Beirut, Lebanon; b Departement of Microbiology, Saint Joseph University, Beirut, Lebanon; c Departement of Sciences, Saint Joseph University, Beirut, Lebanon

## Abstract

The aim of this study was to find the best growth conditions of Enterococcus faecalis on a dentinal substrate in order to be used for the development of a complex multispecies endodontic biofilm. Fifty two single rooted extracted human teeth and fifty two dentinal disks were mechanically prepared, sterilized, inoculated with *Enterococcus faecalis* and divided randomly into 8 groups where the substrate, the inoculation technique, the medium type, and the pre-treatment with collagen type I was varied. Bacterial count was evaluated and colonies were counted and confirmed by colony morphology observation on blood agar and Gram staining at 3,7, 14, 21, and 28 days. On day 14 of the culture, the bacterial count showed the highest values in all groups. Root canals and Type 1 collagen pre-treatment and glucose proved to have significant positive effects on the bacterial count compared to dentinal disks and BHI media only. The increase in bacterial count found with the direct inoculation technique was not significantly different from that of the indirect technique.

## Introduction

Endodontic infections entail a dynamic interplay of microorganisms within the intricate anatomical confines of the root canal system [1]. Commencing with the adherence of facultative anaerobes, notably Enterococcus faecalis, to dentinal surfaces, subsequent colonization by obligate anaerobic species follows, fostering the establishment of tenacious biofilm communities [2]. These anaerobic consortia orchestrate a sustained and dysregulated host response characterized by the release of pro-inflammatory cytokines, chemokines, and antimicrobial peptides, thereby initiating a cascade of tissue degradation and regenerative attempts[3]. Ultimately, this pathological process culminates in the formation of periapical lesions and, if unchecked, may potentially lead to systemic repercussions, highlighting the critical need for targeted therapeutic strategies. [4].

Microorganisms benefit from the biofilm mode of growth because it creates three-dimensionally structured communities with fluid channels for the transportation of food, waste, and signal molecules [5]. According to Svensäter and Bergenholtz's theory, biofilm formation in root canals begins sometime after the first invasion of the pulp chamber by planktonic oral microbes following some tissue disintegration [6].

According to Costerton et al. [7], the biofilm is composed of individual cells and microcolonies that are collectively immersed in a highly hydrated, primarily anionic exopolymer matrix. Any surface that possesses a fluid containing nutrients can support the growth of bacterial biofilms. Three essential elements are primarily needed for biofilm formation: bacterial cells, a solid surface, and a liquid medium [4,6].

To comprehend the pathogenic potential of the root canal microbiota and to lay the groundwork for novel disinfection strategies, the biofilm notion must be applied to endodontic microbiology. It is crucial to understand how endodontic therapy methods are stopped by the biofilm created by root canal bacteria. The first phase is to understand how root canal bacteria develop a biofilm that resists endodontic treatment [5], In addition to shielding bacteria from the host's immune system, the biofilm community also increases their resistance to some disinfectants used in infection therapy and root canal irrigation methods [6].

The root canal system, on the other hand, is incredibly complex and protects bacteria from instruments and cleaning solutions through the presence of isthmuses, lateral extensions, apical deltas, lateral canals, and dentinal tubules [8]. The biofilm habitat of the bacteria in the root canal also makes things more challenging [4]. The microbial cells cling to the canal walls while submerged in a self-produced extracellular matrix [5, 9]. Compared to their planktonic state, biofilm cells are much more resistant to the majority of antimicrobials and host defenses

The pursuit of identifying optimal growth conditions for *Enterococcus faecalis* on dentinal substrates stems from a critical gap in the existing body of research [4]. While prior studies have undoubtedly contributed valuable insights into the behavior of this microorganism, a comprehensive understanding of its growth dynamics under varying conditions on dentinal substrates remains elusive [2, 4]. Previous research efforts have predominantly focused on general aspects of *Enterococcus faecalis* behavior or explored its interactions with host tissues in broader contexts [4]. The precise mechanisms governing its proliferation on dentin surfaces have not yet been elucidated. This knowledge gap emphasizes the critical necessity for a dedicated investigation aimed at comprehending the intricate interplay between *Enterococcus faecalis* and dentin substrates. The ultimate aim is to devise more effective strategies for combating endodontic infections [2, 4].

The aim of this study was to find the best growth conditions of *Enterococcus faecalis* on dentinal substrates.

## Methodology

All methods were carried out in accordance with the relevant guidelines and regulations, adhering to the strict guidelines set forth The Ethic Committee of Saint Joseph University of Beirut. (FMD200)

### Sample preparation

In total, 72 teeth were utilized in this study. Specifically, 20 freshly extracted first upper molars were selected for the initial phase of preparing dentin disks, while an additional 52 single-rooted first upper premolars extracted for orthodontic reasons were employed in the second phase, focusing on the preparation of root canals.

### Dentinal disk preparation

Twenty freshly extracted first upper molars for periodontal reasons, without caries were carefully selected for this study. A consent form was obtained from the patients prior to the extraction. The molars’ outer surface was thoroughly cleaned to remove any soft tissue remnants using a 5% NaOCl solution, followed by rinsing with sterile water. The teeth were then placed in plastic molds and secured to the base with putty. To create dentinal disks, a mixture of EpoxyResin® base and hardener, in a 5:1 ratio, was prepared and thoroughly mixed to achieve a consistent, bubble-free homogenous blend. This prepared resin was poured meticulously into the plastic molds, ensuring complete coverage of the tooth, and left to set at room temperature for 24 h.

Following the curing process, the resin was meticulously extracted from the plastic molds. Subsequently, utilizing an Isomet®2000 precision saw, the resins were sectioned into 2 mm-thick wafers. The chosen disks met the criterion of having the apical third of the root included, resulting in a total of 52 disks. To eliminate any residual resin and attain smooth surfaces, the samples underwent polishing using polishing disks and Metadi® diamond suspension. After polishing, all disks were thoroughly rinsed with water.

To ensure a clean and uncontaminated surface, the disks were treated with a 5% NaOCl solution and a 17% EDTA solution for 1 min each, using small adhesive brushes. Following this treatment, the disks were rinsed for 5 h under running water to remove any remaining solvents completely. As a final step, all samples were autoclaved using, at 120 °C for 20 min. The dentinal disks were then stored in sterile water at 4 °C until use.

### Root canal preparation

Fifty two single rooted first upper premolar extracted for orthodontic reason, were selected, after having the consent of the patients. The presence of only one canal was confirmed by digital radiography. Subsequently the outer surface was cleaned from any inorganic deposit or remaining soft tissue using an ultrasonic device (Piezon® Master 400 EMS Electro Medical Systems SA, Nyon, Switzerland) and NaOCl 5%. Samples were then washed with distilled water and stored in saline solution at room temperature until starting the experiments. The saline solution was renewed 2 times/week. The same practitioner performed all preparation steps. The crowns were removed using a diamond disk (Prodont Holliger, Vence, France) and all roots were shortened to approximately 15 mm in height. - Afterward, all samples were placed in an Ultrasonic bath (Fisher Scientific Inc., Schwerte, Germany) in order to clean them from the debris of cutting. - The canals were enlarged manually using K-file (Micro-Mega, Besançon, France) as follows: 1. A #10 K-File was introduced in the canals reaching the working length (root canal length minus 1mm), to remove the canal content and confirm the absence of any obstacles in the canal. The preparation was continued by using a reciproc R25 till the working length. The canals were flushed with 1 ml of NaOCl 5% using a 30 gauge lateral needle.

As a final step, all samples were autoclaved using, at 120 °C for 20 min. The dentinal disks were then stored in sterile water at 4 °C until use.

### Monobacterial dentinal infection

*Enterococcus faecalis* derived from ATCC 29212 was obtained from the Microbiologic Department and cultured aerobically on blood agar at 35 °C for 48 h. Colonies were then grown in Brain Heart Infusion (BHI) broth at 37 °C for 24 h in a shaker incubator then placed in a static environment for 24 h. Inoculum was prepared in sterile BHI broth and turbidity was set to 0.5 McFarland corresponding to approximately 1.5 × 10^8^ colony forming units per milliliter (CFU/mL). Ten µl of the culture was immediately placed on the dentinal disks in groups 1, 2, 3, and 4 and inoculated in the root canals in groups 5, 6,7 and 8. Dentinal disks and teeth were placed in sterile cups and incubated at 37 °C for 7 d. Four dentinal disks and four root canals were randomly selected for enumeration of *Enterococcus faecalis* directly after inoculation. Inoculum was renewed every day following incubation to ensure maintenance of the culture viability.

Dentinal disks and root canals were divided into 8 groups of 13 samples each, in each group one parameter was changed (Table 1):

**Table 1.** different parameters and conditions changed in each group (Substrate, inoculation technique, pre-treatement, medium, incubation time).

Group	Substrate	inoculation	Pre-treatement	medium	Incubation time
1	Dentinal discs	Direct	Non treated with type 1 coll.	BHI	3, 7,14,21 days
2	Dentinal discs	Direct	treated with type 1 coll.	BHI + sacc.	3, 7,14,21 days
3	Dentinal discs	indirect	Non treated with type 1 coll.	BHI	3, 7,14,21 days
4	Dentinal discs	indirect	treated with type 1 coll.	BHI + sacc.	3, 7,14,21 days
5	Root canals 25 8%	Direct	Non treated with type 1 coll.	BHI	3, 7,14,21 days
6	Root canals 25 8%	Direct	treated with type 1 coll.	BHI + sacc.	3, 7,14,21 days
7	Root canals 25 8%	indirect	Non treated with type 1 coll.	BHI	3, 7,14,21 days
8	Root canals 25 8%	indirect	treated with type 1 coll.	BHI + sacc.	3, 7,14,21 days

The inoculation technique: in groups 1 and 2 10 µl of the inoculum was directly placed on the dentinal disks using a sterile microbrush, in groups 5 and 6 10 µl of the inoculum was directly inoculated in the root canal. In groups 3 and 4 dentinal disks were soaked in 10 ml of the inoculum, and in groups 7 and 8 root canals were soaked in 10 ml of the inoculum.

The pre-treatment technique: in groups 1,3,5, and 7 the dentinal disks were not treated with type 1 collagen, while in groups 2,4,6, and 8 the disks were treated with type 1 collagen.

BHI and BHI + saccarose 5% were used as medium.

### Bacterial count

In groups 1,2,3,4, the *Enterococcus faecalis* bacterial count was evaluated by scraping the surface of the dentinal disk with a sterile microbrush and in groups 5,6,7, and 8 by placing a sterile paper point into each canal for 5 min. Microbrushes and paper points were then placed in 500 µl sterile BHI broth for 15 min. After mixing by vortex, 50 µl of the liquid medium was serially diluted in sterile BHI broth and plated on blood agar. Culture media was placed at 37 °C for 48 h. Colonies were counted and confirmed by colony morphology observation on blood agar and Gram staining.

The counting method on agar involved two different examinators. After the incubation period, agar plates were visually inspected by each examiner independently. The colony-forming units (CFUs) were manually counted using a standard counting grid or area on the agar plates. This process included examining each colony and recording the count separately for consistency.

## Results

### Statistical analysis

Data were analyzed using IBM SPSS Statistics for Windows, version 26 (IBM Corp., Armonk, NY, USA) with a significance level set at 5%. Descriptive statistics for quantitative variables (bacterial count on days 3, 7, 14, 21, and 28) were presented as means ± standard deviations (SD). Normality of the distribution of the quantitative variables was assessed using the Shapiro-Wilk test. Since variables were normally distributed, a repeated measures analysis of variance (ANOVA) with one within-subjects factor (time) and one between-subjects factor (groups) was conducted in order to evaluate significant differences in bacterial count between groups according to time while comparing for main effects; Bonferroni and Tukey post-hoc tests for multiple comparisons were conducted to compare values of within-subjects factor and values of between-subjects factor respectively. In order to assess the exact effect of each parameter on bacterial count on each time, a repeated measures ANOVA with one within-subjects factor (time) and three between-subjects factors (substrate, inoculation, pre-treatment and medium combined) was conducted and five multiple linear regression models were generated.

## Results

**Figure 1.**
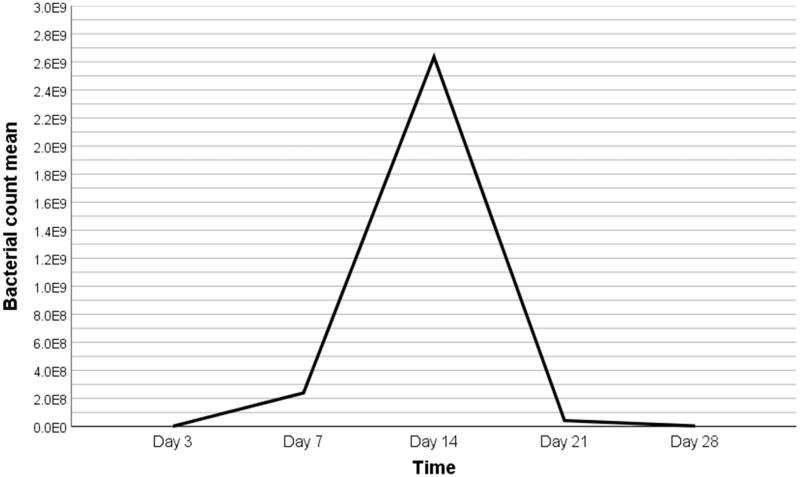
Line chart showing the pattern of progression in bacterial count colonization from day 3 to day 28 (CFU).

A total of 104 culture media equally divided into eight groups were included in the analysis. Results of the comparisons between means of bacterial count according to culture time regardless of groups are shown in Table 2 and Figure 1. Statistically significant differences (P < 0.001) in bacterial count were found between all measurements performed on days 3, 7, 14, 21, and 28 (Table 2); and the highest bacterial count was observed on day 14 (Figure 1).

**Table 2.** Comparisons of the means in bacterial count according to time regardless of groups (N = 104) (CFU)

	Mean ± SD	Minimum	Maximum
*Day 3*	2.08 ± 0.52 (x10^6^)^E^	1.10x10^6^	3.50x10^6^
*Day 7*	2.39 ± 0.50 (x10^8^)^B^	1.30x10^8^	3.70x10^8^
*Day 14*	2.63 ± 0.47 (x10^9^)^A^	1.50x10^9^	3.90x10^9^
*Day 21*	4.20 ± 0.63 (x10^7^)^C^	2.00x10^7^	5.90x10^7^
*Day 28*	4.19 ± 0.75 (x10^6^)^D^	3.10x10^6^	6.30x10^6^

P < 0.05. Different uppercase superscript letters (A > B>C > D>E) indicate statistically significant difference between bacterial count means according to time using Bonferroni multiple comparisons.

Results of the repeated measures ANOVA with one within-subjects factor (time) and three between-subjects factors (substrate, inoculation, pre-treatment and medium combined) that generated five multiple linear regression models are shown in Table 3 and 4.

**Table 3.** Comparisons of bacterial count means according to time and groups (CFU).

		Time					
Grps	Parameters	Day 3	Day 7	Day 14	Day 21	Day 28	*p-*value
1	Dentinal disks. Direct inoculation. Not treated with coll. type 1. BHI.	1.70 ± 0.56(x10^6^)^E^	2.31 ± 0.23(x10^8^)^B^	2.50 ± 0.34(x10^9^)^A^	3.98 ± 0.35(x10^7^)^C^	3.59 ± 0.39(x10^6^)^D^	<0.001*
2	Dentinal disks. Direct inoculation. Treated with coll. type 1. BHI + saccarose 5%.	2.16 ± 0.52(x10^6^)^E^	1.78 ± 0.30(x10^8^)^B^	2.55 ± 0.24(x10^9^)^A^	3.65 ± 0.72(x10^7^)^C^	4.01 ± 0.51(x10^6^)^D^	<0.001*
3	Dentinal disks. Indirect inoculation. Not treated with coll. type 1. BHI.	1.88 ± 0.41(x10^6^)^E^	2.15 ± 0.36(x10^8^)^B^	2.53 ± 0.44(x10^9^)^A^	4.42 ± 0.22(x10^7^)^C^	3.79 ± 0.46(x10^6^)^D^	<0.001*
4	Dentinal disks. Indirect inoculation. Treated with coll. type 1. BHI + saccarose 5%.	1.98 ± 0.41(x10^6^)^E^	2.83 ± 0.66(x10^8^)^B^	2.51 ± 0.57(x10^9^)^A^	4.03 ± 0.57(x10^7^)^C^	4.25 ± 0.49(x10^6^)^D^	<0.001*
5	Root Canal. Direct inoculation. Not treated with coll. type 1. BHI.	2.03 ± 0.44(x10^6^)^E^	2.35 ± 0.40(x10^8^)^B^	2.32 ± 0.34(x10^9^)^A^	3.96 ± 0.38(x10^7^)^C^	4.10 ± 0.35(x10^6^)^D^	<0.001*
6	Root Canal. Direct inoculation. Treated with coll. type 1. BHI + saccarose 5%.	2.41 ± 0.67(x10^6^)^E^	2.49 ± 0.28(x10^8^)^B^	2.98 ± 0.56(x10^9^)^A^	5.11 ± 0.52(x10^7^)^C^	5.59 ± 0.52(x10^6^)^D^	<0.001*
7	Root Canal. Indirect inoculation. Not treated with coll. type 1. BHI.	2.25 ± 0.50(x10^6^)^E^	2.56 ± 0.43(x10^8^)^B^	2.79 ± 0.35(x10^9^)^A^	4.35 ± 0.53(x10^7^)^C^	3.78 ± 0.38(x10^6^)^D^	<0.001*
8	Root Canal. Indirect inoculation. Treated with coll. type 1. BHI + saccarose 5%.	2.23 ± 0.54(x10^6^)^E^	2.66 ± 0.42(x10^8^)^B^	2.89 ± 0.50(x10^9^)^A^	4.11 ± 0.48(x10^7^)^C^	4.45 ± 0.65(x10^6^)^D^	<0.001*
*p-*value		0.016*	<0.001*	0.002*	<0.001*	<0.001*	
Significance between		1 & 6	1 & 2; 1 & 4; 2 & 4; 2 & 5; 2 & 6; 2 & 7; 2 & 8; 3 & 4; 3 & 8;	5 & 6; 5 & 8	1 & 6; 2 & 3; 2 & 6; 2 & 7; 3 & 6; 4 & 6; 5 & 6; 6 & 7; 6 & 8	1 & 4; 1 & 6; 1 & 8; 2 & 6; 3 & 6; 3 & 8; 4 & 6; 5 & 6; 6 & 7; 6 & 8; 7 & 8	

* P < 0.05. Different uppercase superscript letters (A > B>C > D>E) indicate statistically significant difference between bacterial count means in every group according to time using Bonferroni multiple comparisons tests with an adjusted P < 0.01.

**Table 4.** Multiple linear regression models showing effects of each parameter on bacterial counts on days 3 to 28 (CFU).

Model	Parameter	Unstandardized coefficients		Standardized coefficients	p-value
		B	SE	Beta	
Day 3 (x10^6^)	(constant) Substrate	1.265	0.265	–	<0.001*
	Dentinal disc (ref.)	0	–	–	
	Root canal	0.302	0.100	0.282	0.003*
	Inoculation				
	Indirect (ref.)	0	–	–	
	Direct	0.010	0.100	0.009	0.924
	Medium				
	BHI only (ref.)	0	–	–	
	Pre-treatment + BHI + Glucose	0.233	0.100	0.217	0.022*
Day 7 (x10^8^)	(constant) Substrate	1.404	0.238	–	<0.001*
	Dentinal disc (ref.)	0	–	–	
	Root canal	0.248	0.090	0.251	0.007*
	Inoculation				
	Indirect (ref.)	0	–	–	
	Direct	0.313	0.090	0.317	0.001*
	Medium				
	BHI only (ref.)	0	–	–	
	Pre-treatment + BHI + Glucose	0.098	0.090	0.099	0.278
Day 14 (x10^9^)	(constant) Substrate	1.862	0.232	–	<0.001*
	Dentinal disc (ref.)	0	–	–	
	Root canal	0.223	0.088	0.239	0.013*
	Inoculation				
	Indirect (ref.)	0	–	–	
	Direct	0.096	0.088	0.103	0.276
	Medium				
	BHI only (ref.)	0	–	–	
	Pre-treatment + BHI + Glucose	0.196	0.088	0.210	0.028*
Day 21 (x10^7^)	(constant) Substrate	3.510	0.317	–	<0.001*
	Dentinal disc (ref.)	0	–	–	
	Root canal	0.363	0.120	0.290	0.003*
	Inoculation				
	Indirect (ref.)	0	–	–	
	Direct	0.052	0.120	0.041	0.689
	Medium				
	BHI only (ref.)	0	–	–	
	Pre-treatment + BHI + Glucose	0.048	0.120	0.038	0.666
Day 28 (x10^6^)	(constant) Substrate	2.583	0.296	–	<0.001*
	Dentinal disc (ref.)	0	–	–	
	Root canal	0.571	0.112	0.383	<0.001*
	Inoculation				
	Indirect (ref.)	0	–	–	
	Direct	−0.256	0.112	−0.172	0.025*
	Medium				
	BHI only (ref.)	0	–	–	
	Pre-treatment + BHI + Glucose	0.760	0.112	0.509	<0.001*

SE: standard error. *P < 0.05.

At day 3, root canals and media containing Type 1 collagen and glucose significantly increased bacterial count of 0.282x10^6^ (P = 0.003) and 0.217x10^6^ (P = 0.022) respectively, compared to dentinal disks and BHI media without collagen and glucose. No significant effect of the inoculation technique was observed at day 3 (P = 0.924).

At day 7 of the culture, root canal substrates and direct inoculation techniques had significantly increased the bacterial count of 0.251x10^8^ (P = 0.007) and of 0.317x10^8^ (P = 0.001) respectively, compared to dentinal disk substrates and indirect inoculation techniques. A positive effect of Type 1 collagen pre-treatment and glucose on bacterial count was noticed (Standardized Beta = 0.099), but this increase was not statistically significant.

At day 14, root canals and Type 1 collagen pre-treatment and glucose proved to have significant positive effects on the bacterial count compared to dentinal disks and BHI media only. However, despite that the direct inoculation technique increased the bacterial count compared to the indirect technique, this effect was not statistically significant (P = 0.276).

At day 21 of the culture, only root canals proved to have a statistically significant positive effect on the bacterial count compared to dentinal disks. The positive effects on bacterial count of the direct inoculation technique and Type 1 collagen and glucose medium compared to the indirect inoculation technique and BHI only medium proved to be statistically non-significant.

At day 28, both root canal substrate and media with Type 1 collagen and glucose had significant positive effects on bacterial count compared to dentinal disks and BHI media only. In contrast, direct inoculation technique had a significant negative effect on bacterial count compared to the indirect technique at day (Table 4) 28.

## Discussion

The aim of this study was to find the best growth conditions of *Enterococcus faecalis* on dentinal substrates.

The commonly used bacterial strain in endodontic biofilm model systems is *Enterococcus faecalis* [4]. With a reported incidence of over 90% in certain publications, this species has frequently been isolated from teeth with persistent apical lesion that have undergone root canal treatment [2, 4, 12]. It has been demonstrated that *Enterococcus faecalis* can survive hostile circumstances such an alkaline environment and prolonged fasting [12, 13].

Even when cleaned with sodium hypochlorite or treated with calcium hydroxide, it has the capacity to extensively enter dentinal tubules and create biofilms on the root canal wall [14]. *Enterococcus faecalis* has long been regarded as a significant pathogen in endodontology due to the characteristics of the species that enable it to persist in unfavorable environmental conditions and its association with post-treatment disease as revealed by epidemiological research [4, 11]. The likely outcome of this is that there are numerous research examining the effectiveness of a treatment on a monospecies *Enterococcus faecalis* biofilm [4].

Although using human dentine as a substrate in the formation of biofilm is the most rational option [4], extracted teeth typically come from many people of various ages, bringing diversity in the composition and structure of the dentine In their study of *Enterococcus faecalis* adhesion on young and old human root canal dentine, Ozdemir et al. found that the bacterial growth in the "old dentine" samples was more obvious and thicker [15]. When nonbiological materials are used as the substrate, there is less variation in structure and composition, allowing for the standardization of this parameter, but there is no reflection of the in vivo situation [16]. In addition access to the entire canal is required in order to sample it for microbiological analysis. Thus, before bacterial growth, root canals are mechanically prepared. The structure of the canal wall is changed as a result of canal preparation, resulting in the formation of a smear layer [4]. This smear layer blocks the dentinal tubuli and differs in structure and content of the dentine, on which an in vivo biofilm forms [4, 17].

Type 1 collagen and glucose had significant, positive effects on bacterial count in most of the groups. It has been demonstrated that the chemical composition of the substrate affects both the initial attachment of bacteria and the structural organization of the biofilm [1, 2]. These organic coatings, also known as conditioning films, have been proven to modify the surface characteristics of the material and have an impact on microbial attachment [18]. In order to support initial adhesion and bacterial growth, preconditioning of the substratum was done prior to inoculation in four of the groups. During steam sterilization of the sample, high pressures and temperatures cause denaturation of hydrated proteins, which alters the collagen's physical structure [19], and in this study Wiegand et al. showed that as opposed to newly split dentine samples, autoclaved dentine samples showed around a third less bacterial adherence. Collagen coating could therefore increase bacterial adherence following steam sterilization [20].

When compared to development in more nutrient-rich media, *Enterococcus faecalis* cells cultured in phosphate-buffered saline showed improved adhesion to dentine. Saline solution, however, does not accurately represent the in vivo situation [4]. Therefore we used Brain Heart infusion broth and added 5% glucose in four groups. It should be noted that no laboratory technique can accurately replicate the nutritional conditions that the bacteria in the root canal system need to thrive and develop [21]. Since the majority of endodontic biofilm model systems seek to replicate a primary root canal infection, the often employed, extremely nutritive general growth media, which offers a source of both carbs and proteins, appears to be an appropriate option for biofilm growth [22].

Several studies looked into how the age of a biofilm affected the efficiency of an antimicrobial treatment and they showed that all the therapies generally work less well against more developed biofilms [23–25].

In the study of De Meyer et al. (2017) *Enterococcus faecalis* and *Streptococcus mutans* were cultivated in a dual-species biofilm, and 48, 72, and 168 hour incubation durations were evaluated. When cells were recovered via culture, there was no discernible difference in the quantity between the 48- and 72-hold biofilms; however, after 168 hours of incubation, significantly fewer cells were recovered [16]. Growing *Enterococcus faecalis* biofilms in a microtitre plate, Seneviratne et al. found that cell recovery reached its peak after 72 hours and began to drop after 7 days [26]. Shen et al. found that the thickness of multispecies biofilms containing *Enterococcus faecalis* developed on collagen-coated hydroxyapatite disks was less than that of biofilms that were 3-weeks-old and 6-weeks- old [27].

## Conclusion

Considering our study's limitations, it is noteworthy that the highest bacterial count for each group was observed on day 14 of the culture. In contrast to dentinal disks and BHI media alone, root canals, Type 1 collagen pre-treatment, and BHI + 5% glucose exhibited significant positive effects on the bacterial count.

To further emphasize the practical implications of these observations, we recommend that future research explore interventions or treatments targeting the identified influential factors, such as root canal conditions, collagen pre-treatment, and glucose supplementation. These factors could potentially be manipulated to modulate bacterial counts, offering new avenues for biofilm control and management in endodontic contexts.
